# Endogenously elevated bilirubin modulates kidney function and protects from circulating oxidative stress in a rat model of adenine-induced kidney failure

**DOI:** 10.1038/srep15482

**Published:** 2015-10-26

**Authors:** Ai-Ching Boon, Alfred K. Lam, Vinod Gopalan, Iris F. Benzie, David Briskey, Jeff S. Coombes, Robert G. Fassett, Andrew C. Bulmer

**Affiliations:** 1 Heart Foundation Research Centre, Menzies Health Institute Queensland, Griffith University, Gold Coast, Australia; 2 School of Medicine and Cancer Molecular Pathology, Menzies Health Institute Queensland, Griffith University, Gold Coast, Australia; 3 Pathology Queensland, Gold Coast University Hospital, Gold Coast, Australia; 4 Department of Health Technology & Informatics, The Hong Kong Polytechnic University, Hong Kong; 5 School of Human Movement and Nutrition Sciences, University of Queensland, St Lucia, Australia

## Abstract

Mildly elevated bilirubin is associated with a reduction in the presence and progression of chronic kidney disease and related mortality, which may be attributed to bilirubin’s antioxidant properties. This study investigated whether endogenously elevated bilirubin would protect against adenine-induced kidney damage in male hyperbilirubinaemic Gunn rats and littermate controls. Animals were orally administered adenine or methylcellulose solvent (vehicle) daily for 10 days and were then monitored for 28 days. Serum and urine were assessed throughout the protocol for parameters of kidney function and antioxidant/oxidative stress status and kidneys were harvested for histological examination upon completion of the study. Adenine-treated animals experienced weight-loss, polyuria and polydipsia; however, these effects were significantly attenuated in adenine-treated Gunn rats. No difference in the presence of dihydroadenine crystals, lymphocytic infiltration and fibrosis were noted in Gunn rat kidneys versus controls. However, plasma protein carbonyl and F_2_-isoprostane concentrations were significantly decreased in Gunn rats versus controls, with no change in urinary 8-oxo-7,8-dihydro-2′-deoxyguanosine or kidney tissue F_2_-isoprostane concentrations. These data indicated that endogenously elevated bilirubin specifically protects from systemic oxidative stress in the vascular compartment. These data may help to clarify the protective relationship between bilirubin, kidney function and cardiovascular mortality in clinical investigations.

Chronic kidney disease (CKD) is a strong and independent risk factor for cardiovascular disease, which is associated with systemic inflammation and oxidant stress^1^,^2^. Many clinical studies indicate a negative relationship between circulating bilirubin concentrations and the incidence/progression of CKD as well as related mortality in patients on chronic dialysis^3^,^4^,^5^. A low serum bilirubin concentration is associated with accelerated progression of CKD over an ~8 year follow-up period indicating that bilirubin could be an independent predictor of CKD progression^6^. Oda *et al.* also demonstrated that hyperbilirubinemic (>1.24 mg/dL) patients had a reduced incidence of end stage kidney disease^7^ and that mildly elevated serum bilirubin concentrations (>0.8 mg/dL) are associated with improved estimated glomerular filtration rate (eGFR)^8^. A negative correlation between serum total bilirubin concentrations and common carotid intima media thickness was reported in children with end stage kidney disease who had undergone kidney transplantation and peritoneal dialysis, suggesting bilirubin may protect from vascular complications secondary to CKD^9^. Bilirubin is now regarded as an in important multi-point inhibitor of atherosclerosis^10^,^11^ and may protect from vascular damage, via its antioxidant properties thus protecting from free radical induced damage to lipids and proteins^12^,^13^,^14^.

Gilbert’s syndrome (GS) is a benign condition of unconjugated hyperbilirubinaemia (≥17.1 μM) occurring in 5–10% of the general population^15^. Additional TA nucleotide insertions within the promoter region of uridine diphosphate glucuronosyl transferase 1A1 (UGT1A1*28), represents one possible cause of this condition, which significantly reduces hepatic bilirubin conjugation and thus excretion^16^. Hyperbilirubinemic end stage kidney disease patients with the UGT1A1*28 genotype undergoing hemodialysis are protected from cardiovascular events^17^ and all-cause mortality^3^. Possible mechanisms are proposed to explain the protective effect of bilirubin in clinical studies including antioxidant, anti-inflammatory and anti-apoptotic effects, suggesting that it could represent a potential therapeutic compound to reduce the prevalence of CKD or cardiovascular disease^10^. However, only a small number of experimental trials have investigated the protective effects of exogenous bilirubin in response to injury in animal studies^18^,^19^,^20^. For example, exogenous bilirubin administration before and during ischemia-reperfusion injury (IRI) significantly reduces vascular resistance, improves urine output, tubular function and GFR in the isolated, perfused rat kidney compared to control treatment^18^. A later study showed that cortical proximal tubule histology was preserved in bilirubin-treated animals before and during IRI^19^. In addition, Barabas *et al.* demonstrated a protective effect of bilirubin against cisplatin nephrotoxicity in the hyperbilirubinaemic Gunn rat model. Cisplatin-treated Gunn rats maintained normal kidney function and demonstrated significantly preserved histology of the proximal tubule when compared to cisplatin-treated controls^20^. In another study, administration of bilirubin markedly reduced kidney injury markers and improved afferent arteriolopathy, tubulointerstitial fibrosis, and tubular injury in cyclosporine-induced nephropathy in rats^21^. Indeed, a reduced oxidative stress and inflammatory status are reported in hyperbilirubinemic individuals, suggesting bilirubin may be a physiologically important endogenous antioxidant that might prevent glomerular dysfunction and vascular complications^3^,^17^. However, protective ‘antioxidant’ effects in animal models of kidney pathology have not yet been reported.

Adenine phosphoribosyltransferase (APRT) deficiency is a rare autosomal recessive inherited disorder of purine metabolism that usually manifests as 2,8-dihydroxyadenine (DHA) nephropathy due to excessive DHA crystal deposition in the tubular lumen and interstitium of the kidney, leading to inflammation and severely impaired renal function^22^. Patients with DHA nephropathy typically have recurrent nephrolithiasis, which can lead to end stage kidney disease with frequent recurrence in the kidney allograft documented on post-transplant biopsies^22^,^23^. Administration of adenine to rats mimics this condition, as reported previously in the literature^24^,^25^,^26^. This is the first study to investigate the potential kidney protective effects of endogenous hyperbilirubinaemia (Gunn rat) in a model of adenine-induced tubulo-interstitial nephritis.

## Results

### Effects of adenine and methylcellulose (vehicle) administration on body weight

**Figure 1:**
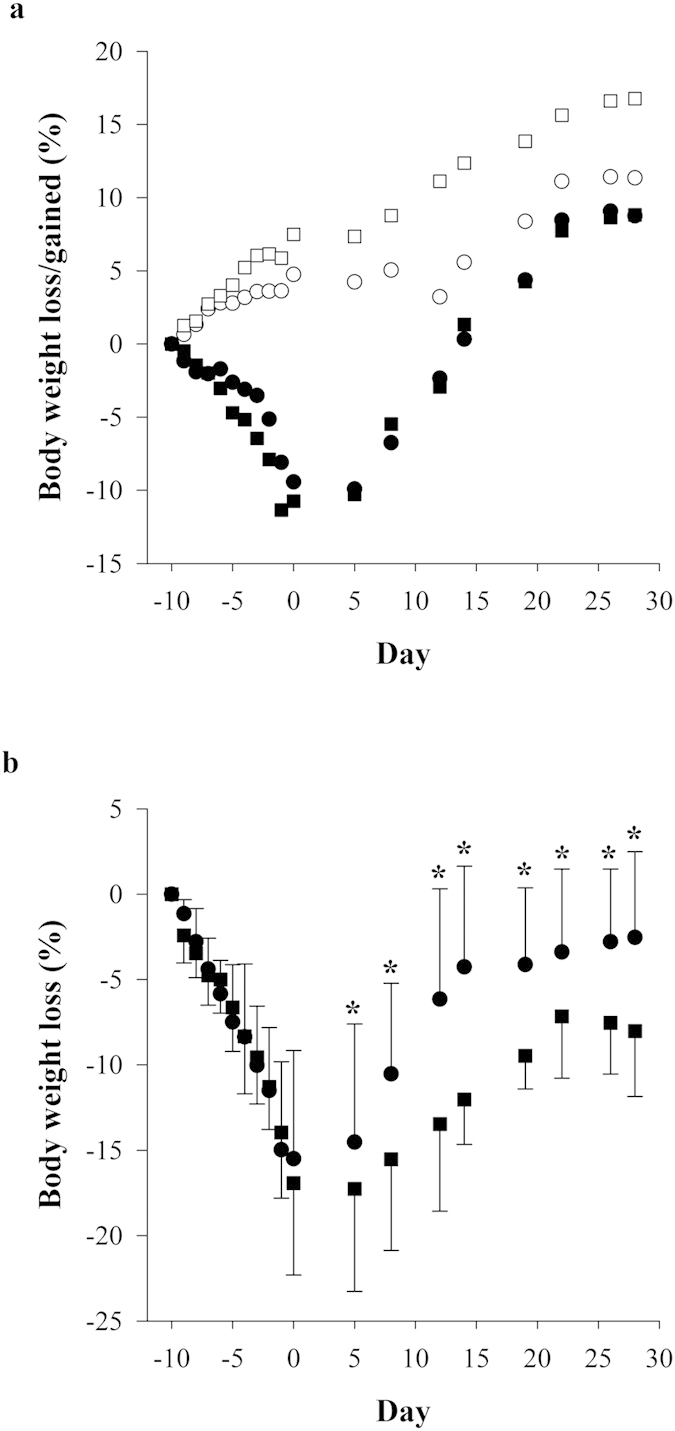
Effects of adenine-induced tubulo-interstitial injury on body weight. (**a**) Percentage body weight loss or gain (from day −10) in animals during adenine and methylcellulose treatment for 10 days, followed by 28 days of observation (•, GA, n = 12; ■, WA, n = 12; ○, GC, n = 9; □, WC, n = 6). Error bars have been removed to improve clarity of this graph. (**b**) Relative body weight loss of adenine treated animals (GA/WA) versus their methylcellulose controls (GC/WC) during adenine and methylcellulose treatment for 10 days, followed by 28 days of observation. Data are expressed as the mean ± standard deviation. *P > 0.05 between the adenine-treated groups.

Adenine or methylcellulose solvent control was orally administered daily for 10 days. During this time, body weight of the adenine-treated animals significantly decreased when compared to methylcellulose treated Gunn (GC) and Wistar (WC) rats (Fig. 1a). No significant difference in weight loss was observed between adenine-treated Gunn and Wistar rats during adenine treatment. However, when body weight loss of adenine-treated animals was expressed relative to the respective control animals’ weight (Fig. 1b), more body weight was regained in Gunn rats treated with adenine (GA) after day +5 of monitoring (*P *< 0.05; versus Wistar rats treated with adenine [WA]).

### Urine output and water consumption

**Figure 2:**
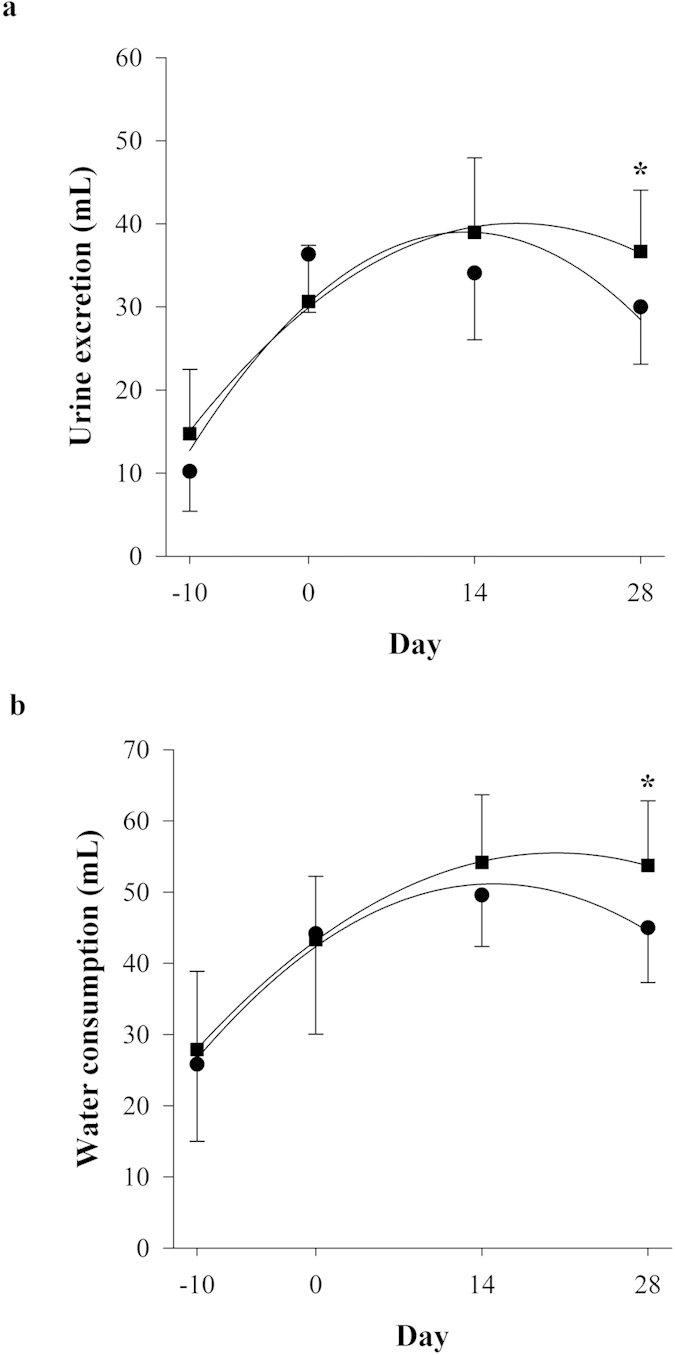
Effects of adenine-induced tubulo-interstitial injury on urine output and water consumption. Urine output (**a**) and water consumption (**b**) in animals treated with adenine for 10 days and then monitored for 28 days (•, GA; ■, WA; n = 12 per group). Data are expressed as a mean ± standard deviation. *P < 0.05 between GA and WA groups.

Solvent (vehicle) control and adenine treated animals were assessed for markers of kidney function/damage. All subsequent parameters for solvent-treated animals are presented in the supplementary material to confirm the absence of kidney disease in the vehicle only treated animals (i.e GC and WC). Urine output and water consumption of adenine-treated animals indicated the development of polyuria from day 0 to 28 after induction of tubulo-interstitial inflammation. Polyuria was accompanied with polydipsia in these animals (Fig. 2). Significant (*P *< 0.05) reductions in urine output and water intake were observed in GA, compared to WA, at day 28. Polyuria and polydipsia were not noted in methylcellulose only treated animals (Figure S1).

### Serum and urinary parameters of kidney function

**Figure 3:**
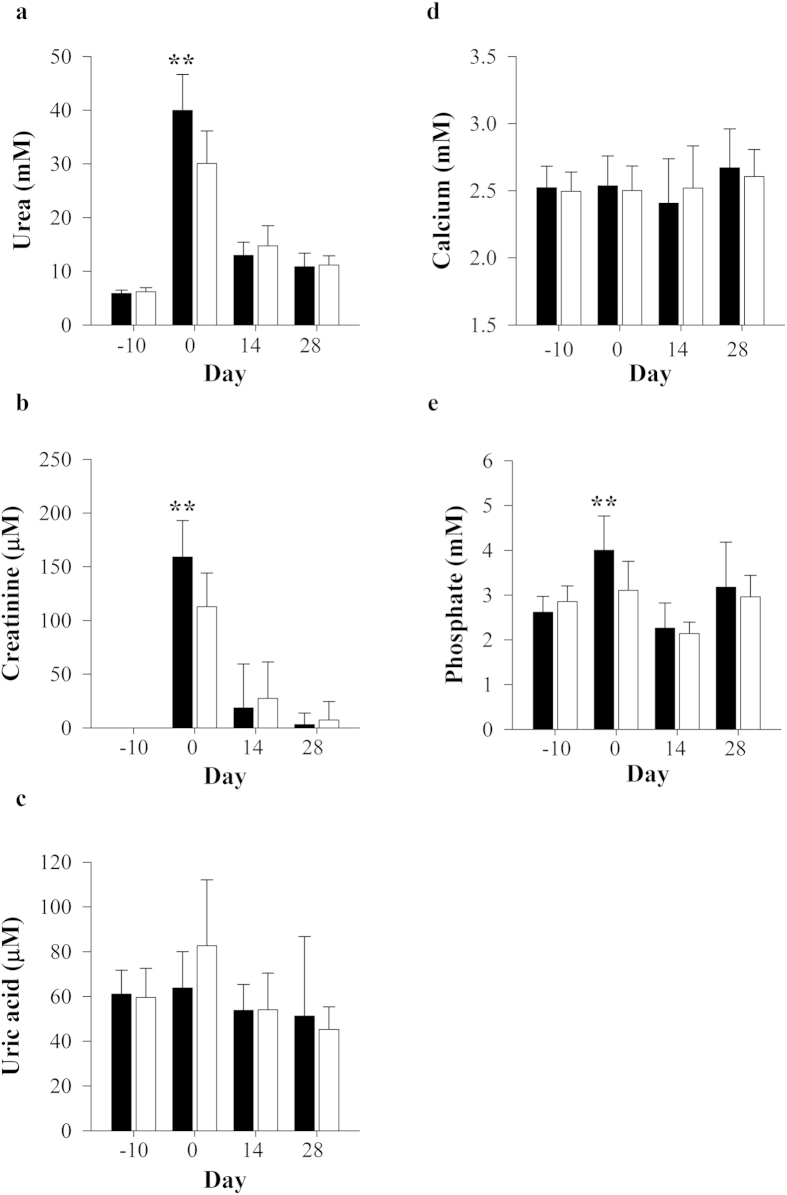
Effects of adenine-induced tubulo-interstitial injury on blood biochemistry. Serum (**a**) urea, (**b**) creatinine, (**c**) uric acid, (**d**) calcium and (**e**) phosphate concentrations were measured in adenine-treated Gunn (GA; black bars) and Wistar rats (WA; white bars; n = 12 per group) at day −10, 0, 14 and 28. Data are expressed as mean ± standard deviation. *P < 0.05, **P < 0.01 between GA and WA groups.

**Figure 4:**
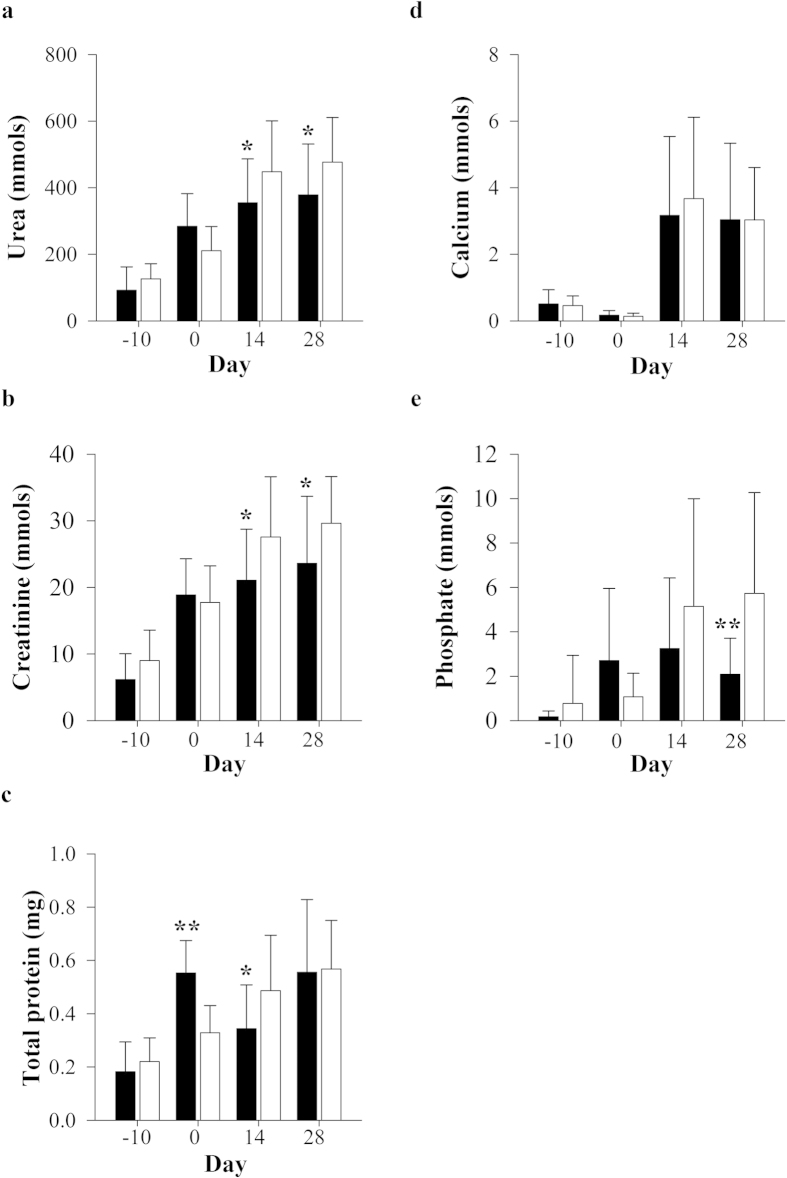
Effects of adenine-induced tubulo-interstitial injury on urine biochemistry. Urinary excretion (24 hour) of (**a**) urea, (**b**) creatinine, (**c**) total protein, (**d**) calcium and (**e**) phosphate were measured in adenine-treated Gunn (GA; black bars) and Wistar rats (CA; white bars; n = 12 per group) at day −10, 0, 14 and 28. Data are expressed as mean ± standard deviation. *P < 0.05, **P < 0.01 between the GA and WA groups.

Serum and urine biochemistries of adenine-administered (Table S1) and vehicle-control animals (Table S2) were examined at baseline and throughout the observation period of 28 days. No significant (*P *< 0.05) differences in albumin, total protein or glucose were noted between the groups. Gunn rats had reduced total cholesterol and greater triglyceride concentrations versus controls (Table S1). Serum liver enzyme activities were elevated in adenine-treated groups on day 0, with increased ALT and reduced GGT activities demonstrated in GA compared to WA at day 10. Serum urea and creatinine concentrations were mildly increased in Gunn rats compared to Wistar controls after adenine treatment for 10 days (i.e. day 0; Fig. 3a and b; *P *< 0.01). However, all concentrations returned to baseline values after 14–28 days. There were no changes in serum uric acid and calcium concentrations between adenine treated groups (Fig. 3c,d). A mild, yet significant (*P *< 0.01) increase in serum phosphate concentrations was also observed in GA at day 0 (Fig. 3e). Total urinary excretion of urea, creatinine and total protein were increased after adenine dosing at day 0 in all animals and generally continued to increase over the observation period of 28 days (Fig. 4a–c). Urea and creatinine output were significantly reduced in GA at day 14 and 28 (*P *< 0.05, Fig. 3a,b). Similarly, GA had significantly reduced total protein and phosphate excretion at day 14 and 28, respectively (Fig. 3c,e).

**Figure 5:**
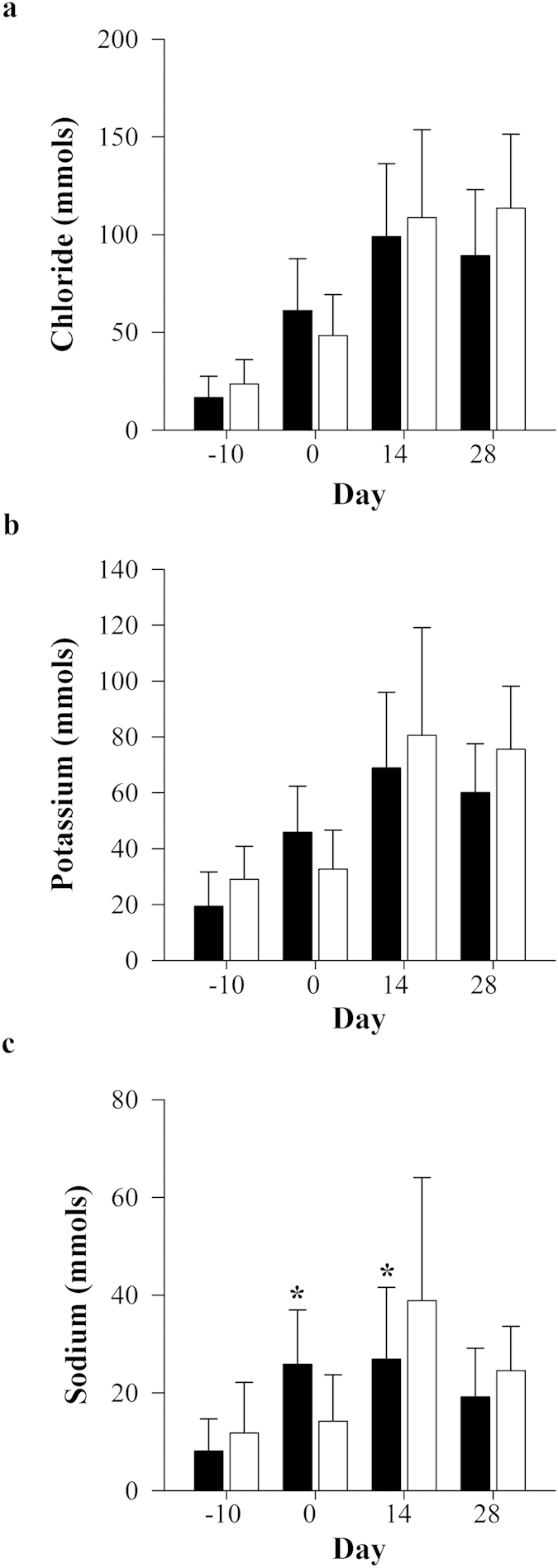
Effects of adenine-induced tubulo-interstitial injury on 24 hour urinary electrolyte excretion. Urinary excretion of (**a**) chloride, (**b**) potassium and (**c**) sodium were measured in adenine-treated Gunn (GA; black bars) and Wistar rats (WA; white bars; n = 12 per group) at day −10, 0, 14 and 28. Data are expressed as mean ± standard deviation. *P < 0.05 between the groups.

Urinary excretion of electrolytes progressively increased in animals after adenine-treatment at day 0 (Fig. 5). Adenine-treated Gunn rats experienced increased urinary excretion of sodium at day 0 (*P *< 0.05); however, was significantly reduced at day 14 when compared to controls (*P *< 0.05; Fig. 5c).

### Antioxidant/oxidative stress status

**Figure 6:**
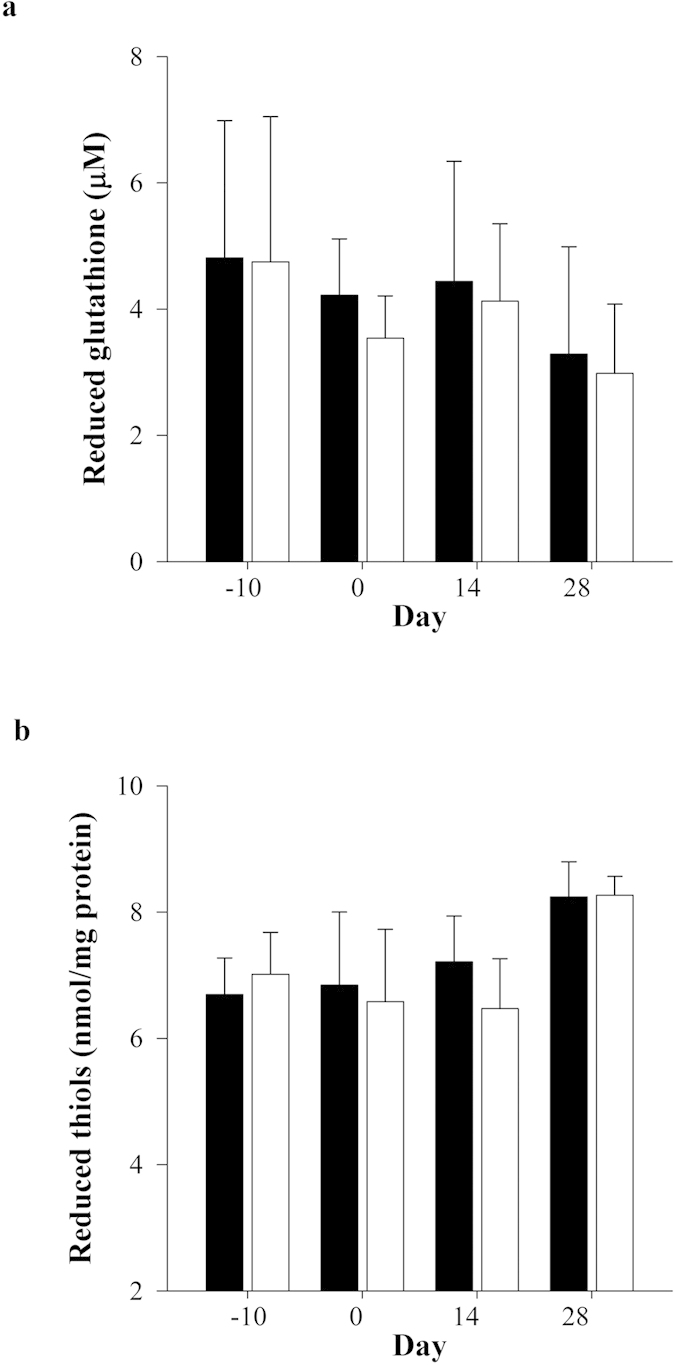
Effects of adenine-induced tubulo-interstitial injury on antioxidant status. (**a**) Reduced glutathione and (**b**) thiol concentrations were measured in adenine-treated Gunn (GA; black bars) and Wistar rats (WA; white bars; n = 12 per group) at day −10, 0, 14 and 28. Data are expressed as mean ± standard deviation.

Direct and total bilirubin concentrations were significantly elevated in Gunn rats versus controls throughout the protocol (Table S3 and S4). However, reduced thiols, when expressed relative to protein, and reduced glutathione (a thiol containing antioxidant) were not significantly different between WA and GA at all-time points (Fig. 6a; Table S3 and S4).

**Figure 7:**
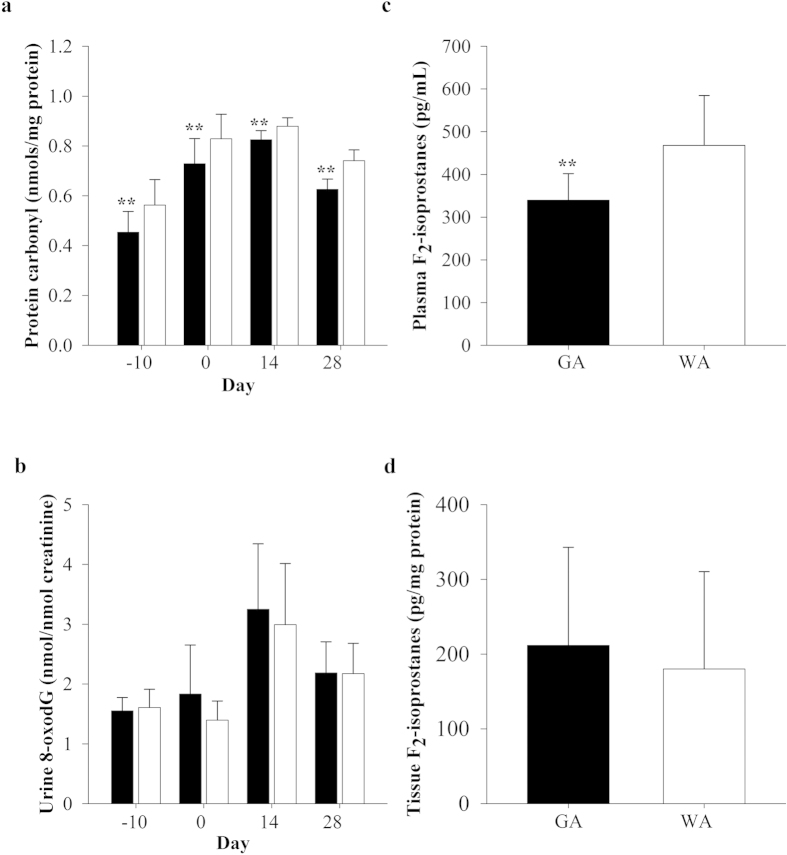
Oxidative damage in the vascular compartment and kidney tissue. (**a**) plasma protein carbonyl and (**b**) urinary 8-oxodG concentrations were measured in adenine-treated Gunn (GA; black bars) and Wistar rats (WA; white bars; n = 12 per group) at day −10, 0, 14 and 28. F_2_-isoprostane concentrations were measured in (**c**) plasma and (**d**) kidney tissue at day 28. Data are expressed as mean ± standard deviation. **P < 0.01 between the GA and WA groups.

No difference in GSSG concentrations and the GSH:GSSG ratio were noted between the groups at any time-point (Table S3). Furthermore, plasma protein carbonyl concentrations were significantly (*P < *0.05) decreased in GA compared to WA throughout the protocol (Fig. 7a). Plasma F_2_-isoprostane concentrations were also significantly (*P < *0.01; Fig. 7c) decreased in GA compared to WA at day 28. No difference was observed in kidney F_2_-isoprostane content between the groups (Fig. 7d). Furthermore, urinary 8-oxo-7,8-dihydro-2′-deoxyguanosine (8-oxodG) concentrations did not differ between the groups at any time-point (Fig. 7b).

### Histopathology

**Figure 8:**
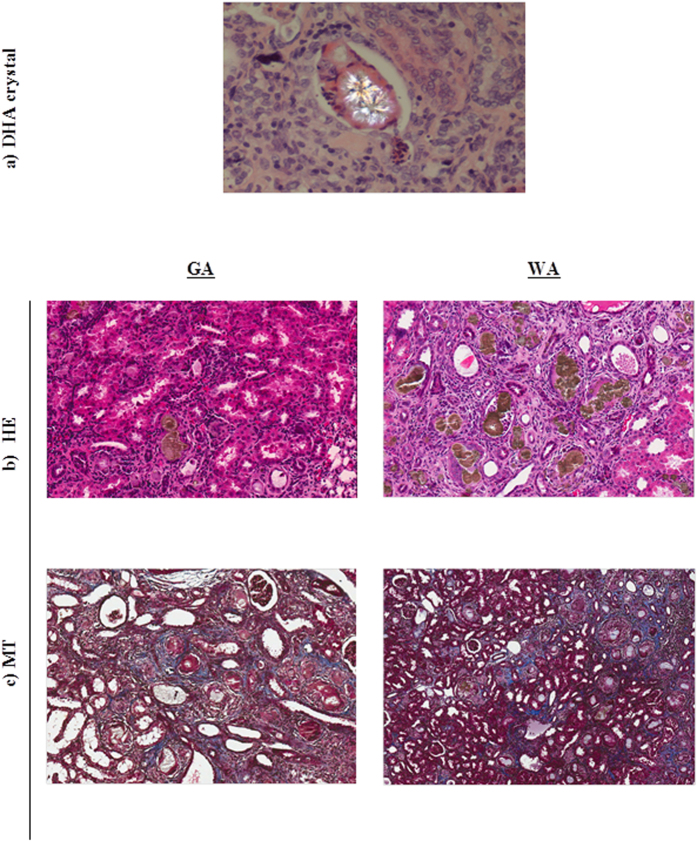
Effects of adenine-induced tubulo-interstitial injury on kidney morphology. (**a**) DHA crystal deposition in a kidney section was highlighted using polarized light (staining by hematoxylin-eosin; HE). Histopathological examination of kidney sections stained with (**b**) HE and (**c**) Masson’s Trichrome (MT) in animals treated with adenine (GA and WA) at day 28.

There were no statistical differences in the distribution of histological scoring of kidneys after 28 days when compared the adenine-treated groups (Table S5). Dihydroadenine crystals were detected in the hematoxylin-eosin (HE) stained animal kidneys treated with adenine and were highlighted using polarised light (Fig. 8a,b). Representative slides revealed similar infiltration of foreign body giant cells reactive to the crystal in the kidney interstitium of animals treated with adenine (Fig. 8b). Lymphocytic infiltration and interstitial calcification were detected via HE staining (Fig. 8a) and interstitial fibrosis was observed in Masson trichrome stained sections in adenine treated animals (Fig. 8c). The kidney morphology of animals treated with methylcellulose stained with HE appeared unremarkable (Figure S2), the scoring of which is presented in Table S6.

## Discussion

The main findings of this study were that hyperbilirubinaemic Gunn rats, when treated with adenine, experienced reduced polyuria and polydipsia and concomitantly regained and maintained their body mass compared to normo-bilirubinaemic animals. Although hyperbilirubinaemia did not improve systemic markers of kidney function, Gunn rats evidenced an improved circulating antioxidant status (total bilirubin) and decreased circulating oxidative stress biomarker concentrations (protein carbonyl and F_2_-isoprostanes) compared to controls after adenine administration. Decreased concentrations of circulating/systemic markers of oxidation were not accompanied by similar changes in markers that reflect cell-based oxidative stress (i.e. kidney F_2_-isoprostanes and urinary 8-oxodG) suggesting that bilirubin’s protective effects were largely confined to the vascular compartment. This is the first study to demonstrate that bilirubin may protect circulating proteins and lipids in blood from adenine-induced oxidative stress *in vivo*, providing further insight into protection from vascular injury during CKD in individuals with elevated bilirubin concentrations (e.g. GS).

Adenine is metabolised to 2,8-dihydroxyadenine (DHA), which is not readily excreted by the kidney, resulting in the deposition of DHA crystals in kidney tubules. This deposition induces chronic kidney disease due to interstitial inflammation, tubular damage and fibrosis^25^,^27^. Adenine-induced chronic kidney failure in this rat model mimics the clinical condition of human chronic kidney failure^25^,^27^,^28^,^29^ and several studies have reported a reduction in body weight in adenine-fed animals^27^,^28^. Terai *et al.* administered adenine (100 mg/rat) and methylcellulose daily for 12 days^28^, which was accompanied by significant body mass reduction compared to solvent control treated animals. Body mass reduction upon adenine feeding is associated with reduced food consumption (approximately one half versus controls)^28^ and azotemia as documented here. Body weight decreased similarly in adenine treated Gunn and Wistar animals in this study (10 days of adenine administration; 300 mg/kg/day). Compared to WA, GA regained greater body mass from day +5 of monitoring indicating that they may have experienced improved food/water intake and/or kidney function during adenine induced interstitial nephritis.

Polyuria and polydipsia are the most common and earliest clinical manifestations of CKD. Adenine treated animals increased their urine output and water intake in association with impaired kidney function after adenine treatment. However, GA experienced a significant improvement in this marker of kidney function compared to WA at day 28. Anti-diuretic hormone (ADH) assists in controlling the body’s water and electrolyte balance by influencing the water excretion by the kidneys^30^. When blood osmolality increases, ADH secretion is stimulated and causes the insertion of water channels into cells lining the collecting ducts, allowing water reabsorption to occur. Tubular reabsorption of sodium is dependent on sympathetic tone and angiotensin II acting via the effects of ADH and aldosterone to conserve water and sodium^31^. Decreased urine output in GA suggests improved reabsorption of water in GA and that Gunn rat kidneys may be able to respond to ADH secretion by increasing water reabsorption in the collecting tubule. This hypothesis agrees with published data indicating that exogenous bilirubin (10 μM) significantly reduces urine production and urinary excretion of sodium, suggesting improvement in kidney vascular resistance and tubular function in an ischemia-isolated, perfused rat kidney *ex vivo*^18^.

Impaired kidney function is reflected by the kidneys’ tubules ability to maintain plasma urea and creatinine clearance^32^. Kidney dysfunction was manifested by a significant elevation of serum urea and creatinine concentrations in adenine-treated animals at day 0 versus baseline (−10). However, the concentrations of these analytes improved similarly during the monitoring period from day 14 and 28. Adenine-treated animals showed a clear progressive increment in urinary excretion of urea and creatinine; however, these effects plateaued earlier in Gunn rats and were significantly reduced in Gunn rats at day 14 and 28. Our results showed increased proteinuria and phosphate excretion in GA compared to WA at day 0; however, this effect was transient and was significantly reduced compared to Wistar animals at day 14 and 28, respectively. Proposed mechanisms which induce proteinuria include oxidative stress, inflammation and initiation and progression of tubulo-interstitial fibrosis^33^. A recent clinical study demonstrated that IgA nephropathy patients with mildly elevated serum bilirubin concentrations had reduced urinary protein concentration, serum creatinine and improved eGFR^8^. In addition, serum bilirubin concentrations were inversely associated urinary albumin excretion in hypertensive patients^34^ and 24 hour urinary protein excretion in both diabetic and non-diabetic adults^35^. These data suggest that bilirubin could protect from glomerular injury, or improve tubular protein reabsorption after kidney inflammation had been induced. However, our data do not fully support these conclusions and are likely a consequence of the model used in this investigation which induced tubulo-interstitial rather than glomerular disease.

Chronic kidney disease is accompanied by characteristic abnormalities in lipid metabolism, which is reflected by elevated plasma lipid levels in patients with all stages of CKD^36^. It is important to note that Gunn rats (control or treated with adenine) possessed reduced total cholesterol concentrations compared to littermate controls, in agreement with previous findings^37^,^38^. Gunn rats maintained a hypocholesterolemic state throughout kidney disease induction which might indicate the importance of bilirubin induced hypocholesterolaemia, on protection from vascular disease in patients with CKD.

Dihydroxyadenine crystal deposition induces degenerative changes in the kidney tubules and causes kidney dysfunction with decreased concentrations of serum calcium and increased concentrations of phosphate^24^,^28^,^39^. Although serum calcium concentrations did not change in adenine-treated animals during the experimental period; a significant increase in urinary excretion of calcium was observed on day 14 and 28 in both groups. These results suggest dysfunctional calcium reabsorption and/or up-regulation of bone reabsorption leading to calcium excretion^40^. Although serum phosphate concentrations were elevated in GA at day 0 and normalised thereafter, reabsorption of filtered phosphorus (inferred from urinary phosphate output) was significantly improved in GA when compared to WA at day 28. Increased serum phosphate and calcium concentrations lead to vascular calcification which is an important risk factor for CKD in hemodialysis patients, and is associated with increased risk of atherosclerosis, ischemic heart disease, and vascular stiffening^40^. Previous studies have demonstrated an inverse relationship between bilirubin concentrations and vascular calcification^41^,^42^. Although our results do not support these findings, it is possible that mildly elevated bilirubin may protect from DHA-induced oxidative stress in a clinically relevant condition such as APRT deficiency.

Despite the lack of significant differences in kidney function parameters (i.e. serum urea and creatinine concentrations) between the Wistar controls and Gunn rats-treated with adenine, potentially important differences in antioxidant defences and oxidative stress biomarkers were noted between the two groups. As expected, Gunn rats possessed significantly elevated serum total bilirubin concentrations throughout the experimental period. It is presumed that the protective effects of bilirubin, shown in clinical studies, on kidney dysfunction are due to bilirubin’s antioxidant properties^18^,^19^,^21^. Importantly, the concentrations of GSH and total reduced thiols were elevated (albeit not significantly so) in GA compared to WA at day 0 and 14.

These data are in agreement with previous studies including individuals with benign hyperbilirubinemia who also possess significantly elevated GSH and SH concentrations compared to controls, the concentration of which was positively correlated with unconjugated bilirubin (UCB) concentrations^37^,^43^. The glutathione antioxidant system is regarded as an important marker of oxidative stress in chronic kidney disease^44^,^45^. Previous studies have shown that the uraemic state is associated with low concentrations of GSH and reduced thiols (SH), which continue to decrease with the progression of kidney failure in dialysis patients^45^. Our findings, therefore, may indicate that bilirubin plays an important role in maintaining circulating thiol status providing an important pool of antioxidant capacity, which can protect proteins and lipids from oxidation by oxidants produced during inflammation, namely hypochlorous acid produced from myeloperoxidase (MPO)^46^. The importance of MPO-catalysed protein and lipid adduct formation has been considered an important mechanistic link between oxidation, atherosclerosis and kidney disease^47^, which bilirubin effectively inhibits^46^. A positive relationship between thiol oxidation, protein carbonylation and kidney dysfunction was reported in CKD patients who underwent dialysis^48^. Moreover, dialysis patients have greater plasma MPO activities and concentrations^49^ in addition to increased protein carbonyl concentrations^50^, which are stable biomarkers of protein oxidation and damage^44^. Individuals with elevated bilirubin concentrations have decreased protein carbonyl concentrations^37^. Therefore, to determine whether elevated bilirubin and thiol/glutathione concentrations could protect against systemic oxidative stress in this model we measured systemic protein carbonyl concentrations. In the present study, GA demonstrated significantly decreased concentrations of protein carbonyls at all-time points compared to WA. Concomitantly, GA showed significantly decreased serum F_2_-isoprostane concentrations, the gold standard biomarker of lipid peroxidation that is associated with inflammation and oxidant stress^51^. These results support the protective effect of bilirubin against protein oxidation and lipid peroxidation in *ex vivo*^52^ and *in vivo*^53^ studies. The effect of bilirubin could be due to direct chloramine quenching^46^, or its thiol preserving capacity, either of which could protect proteins and lipids from MPO- induced oxidation.

In this study, we also report the deposition of bright symmetric crystalline DHA structures in the tubular lumen of adenine-treated animals. These deposits stimulate a local inflammatory response (mostly lymphocyte) and giant cell infiltration which were detected 28 days after adenine administration ceased. Macrophage infiltration triggers the initiation of interstitial fibrosis which results in collagen deposition in the interstitial space^27^,^54^. Lack of clear differences in histological grading (eg. inflammation, calcification and fibrosis) between the adenine-treated groups indicates that elevated bilirubin does not protect the tissue *per se*, from inflammation, calcification and oxidative injury. In contrast to plasma levels of F_2_-isoprostanes, kidney F_2_-isoprostane concentrations were not significantly different between the groups. Furthermore, the renal excretion of the stable DNA oxidation biomarker 8-oxo-7,8-dihydro-2′-deoxyguanosine (8-oxodG), when expressed relative to urinary creatinine concentrations, was not different between the groups. Urinary 8-oxodG is considered to reflect global oxidation of the nucleotide pool, which is largely intracellular, and therefore acts as a whole-body marker of oxidative stress^55^. With contrasting improvements in oxidative damage within the vascular and kidney/organ compartments, these data indicate that bilirubin’s protective effects are focussed within the circulatory compartment where it is bound to albumin, as supported by pharmacokinetic and volume of distribution calculations^56^.

Despite some potentially important findings reported here, it should be noted that this study has several limitations. Administration of adenine sulphate suspension contributed to variability in the pathophysiological sequelae of administration and therefore, our ability to detect statistically significant differences between the groups. Longer-term adenine administration (e.g. <4 weeks) modulates kidney function with less reversibility^25^ and therefore could provide more reliable and thus significant outcomes. We did not measure blood pressure and kidney injury biomarkers including kidney injury molecule-1 and neutrophil gelatinase-associated lipocalin and therefore, we could not determine whether bilirubin’s antioxidant effects, lead to improvements in vascular tone and reactivity.

In conclusion, we have demonstrated that Gunn rats maintain body weight, experience reduced polyuria and polydipsia when compared to Wistar rats after treatment with adenine. Elevated circulating bilirubin concentrations prevented protein and lipid oxidation within the circulatory compartment during kidney inflammation in Gunn rats. However, these data suggest that endogenously elevated bilirubin does not protect from kidney dysfunction (as assessed by accumulation of urea/creatinine) or inflammation, fibrosis/organ oxidative damage. This study suggests that endogenously elevated bilirubin may improve red-ox status and protect from kidney injury-associated vascular damage. Bilirubin protects from cardiovascular events in patients undergoing chronic hemodialysis^3^,^17^. Although bilirubin might not protect from the decrement of kidney function over time, it might protect from endothelial dysfunction, vascular calcification, hypertension, lipoprotein oxidation and dyslipidemia, which are strongly implicated with CKD and contribute to longer term cardiovascular mortality^10^.

## Methods

### Animals

Breeding pairs of heterozygote (genotyped) Gunn rats were purchased from the Rat Research and Resource Center (Columbia, MO, USA). Rats were housed at Griffith University (12-h light: dark cycle, constant temperature (22 °C) and humidity (60%) and had continuous access to standard laboratory food pellets (Speciality Feeds, Glen Forrest, Australia) and fresh water. Male homozygous Gunn rat offspring possess jaundice at birth, were ear-tagged, and housed together with male littermate (non-jaundiced) controls after weaning. Ten week-old male Gunn and Wistar rats were used in this experiment and all procedures were performed in accordance with relevant guidelines and approved by the Griffith University Animal Ethics Research Committee prior to commencement of experimentation (MSC/12/12).

### Oral adenine model

Animals were randomly divided into four groups. Gunn (GA) and littermate Wistar (WA) rats (n = 12 per group) were orally administered adenine in a suspension in 0.5% methycellulose (300 mg adenine sulphate/kg body weight; Sigma-Aldrich, Australia) once daily for 10 days (i.e. induction period) and monitored for 28 days thereafter as tubulo-interstitial nephritis developed^24^,^28^,^29^. Control Gunn (GC; n = 9) and littermate Wistar rats (WC; n=  6) were given 0.5% methylcellulose (mL/kg body weight) daily by oral gavage. Animal body weights were measured daily for 10 days during adenine treatment (induction) and fortnightly thereafter. Blood and urine samples were collected at baseline (−10 days), after induction (day 0) and then fortnightly (days 14 and 28) for biochemical assessment of kidney function. Animals were housed in metabolic cages for 24 hours with free access to food and water. Twenty-four hour urine output/composition and water consumption was collected at day −10, 0, 14 and 28. Urine excretion and water intake volume were measured manually. Approximately 1 mL of blood was collected from the tail tip with brief isoflurane anaesthesia (3% in 100% O_2_; 1–2 L min^−1^) and transferred into serum and EDTA vacutainers. On day 28, all animals were anaesthetised using an intraperitoneal injection of pentobarbital sodium (concentration 60 mg/mL; 100 μL/100 g). A midline laparotomy/thoracotomy was performed, and approximately 5 mL of whole blood was collected using syringe from the thoracic cavity and transferred into serum and EDTA vacutainers. Animals were euthanized by removing the heart. Kidneys were also harvested for histological examination. Kidney tissue was frozen in liquid nitrogen and then crushed using a mortar and pestle and kept at −80 °C for F_2_-isoprostane analysis.

### Sample preparation

Whole blood was centrifuged (Thermo Scientific 5810R, Australia) at 2500 × g for 10 min (4 °C). Serum/plasma and urine aliquots were prepared immediately and stored at −80 °C until analysis. Trichloroacetic acid (10%; ChemSupply, Australia) was added to serum aliquots in 1:1 ratio, which were then vortexed and centrifuged at 2500 × g for 5 min. Supernatant was stored at −80 °C for reduced and oxidized glutathione analysis.

### Biochemistry

Serum samples were analysed for creatinine, uric acid, urea, calcium, phosphate and albumin using a COBAS Integra 400 blood chemistry analyser (Roche Diagnostics, Australia) to monitor the extent of kidney dysfunction. Systemic markers of liver function (alanine aminotransferase, aspartate aminotransferase, γ-glutamyltransferase activity), glucose, lipid parameters (total cholesterol and triglyceride), lipase activity, total protein, total and direct (conjugated) bilirubin were assessed using the same analyser. Cholesterol analyses were conducted using appropriate lipid standards (Calibrator for Automated Systems Lipids) and quality controls (Precinorm Control Clin Chem Multi 1 and 2; Roche Diagnostics, Australia).

Kidney excretion of creatinine, urea, calcium and phosphate were also analysed in urine samples. Total output of the solutes above was calculated from the volume of urine excreted and the concentration of each analyte within each sample. Total protein in urine was quantified using appropriate standards and quality controls (total protein urine quality control; Roche Diagnostics, Australia). Ionic composition of urine (e.g. chloride, potassium and sodium) was assessed using ion-selective electrodes, in addition to calibrators including direct and reference electrolyte solutions (Roche Diagnostics, Australia). All the tests were conducted in duplicate.

### Measurement of reduced and oxidised glutathione concentrations

Reduced (GSH) and oxidised (GSSG) glutathione concentrations were quantified in trichloroacetic acid supernatants of serum using N-ethylmaleimide (NEM) and o-phthalaldehyde (OPA) via a modified method of Hissin and Hilf^57^. The fluorescence intensity of GSSG and GSH were determined using black 96 well plates (Corning, Australia) at 350 nm (excitation) and 420 nm (emission) with a Fluoroskan Ascent 96 well plate reader (Thermo Scientific, Australia). The concentrations of GSH and GSSG in the samples were determined in triplicate using external standards ranging from 1.56–50 μM. The co-efficient of variation for this method was 3.4%.

### Measurement of reduced thiol concentrations

Reduced thiol concentrations were measured using 5,5-dithiobis(2-nitrobenzoic acid) (DTNB; Sigma-Aldrich, Australia) reagent according to the method of Hawkins *et al.*^58^. Reduced thiols react with DTNB to generate 5-thio-2- nitrobenzoic acid (TNB), which was quantified in triplicate at 415 nm using a 96-well plate reader (Multiskan FC, Thermo Scientific, Australia). Reduced glutathione (0–0.5 mM) served as an external standard and thiol concentrations were expressed initially in μM and then converted to nmol/mg of protein. The co-efficient of variation for this method was 2.6%.

### Measurement of protein carbonyl concentrations

Detection of protein carbonyl with 2,4-dinitrophenylhydrazine in EDTA plasma was performed using enzyme-linked immunoassay (ELISA) kits (Sapphire Biosciences, Australia) with the above-mentioned 96-well plate reader. Results were expressed in nmols/mg of protein and all analyses were conducted in duplicate. The co-efficient of variation for this assay was 6.8%.

### Measurement of F_2_-isoprostanes

Samples were analysed in duplicate using our previously published method^59^. Total F_2_-isoprostanes were extracted from plasma and kidney tissue after saponification with methanolic NaOH. Samples were spiked with 8-iso-PGF2α-d4 (Cayman Chemicals, USA) as an internal standard and incubated at 42 °C for 60 min. Samples were then acidified (pH 3) with hydrochloric acid, and hexane added and samples, mixed for 10 min before centrifugation. The supernatant was removed and the remaining solution extracted with ethyl acetate and dried under nitrogen. Samples were reconstituted with acetonitrile, transferred into vials with silanised glass inserts and dried. Derivatisation with pentafluorobenzylbromide and diisopropylethylamine and incubation at room temperature for 30 min followed. Samples were then dried under nitrogen before pyridine, Bis(trimethylsilyl)trifluoroacetamide (99%) and trimethylchlorosilane (1%) were added and incubated at 45 °C for 20 min. Finally, hexane was added samples were mixed, then 1μL was injected for analysis using gas chromatography tandem mass spectrometry (Varian, Australia) in negative chemical ionization mode. The coefficient of variation for this assay was 4.5%.

### Measurement of urine 8-oxo-7,8-dihydro-2′-deoxyguanosine

Each sample (urine samples, pooled urine and controls) at −80 °C was thawed to room temperature and analysed for 8-oxo-7,8-dihydro-2′-deoxyguanosine (8-oxodG) according to our previously published method^60^. Samples were vigorously mixed, sonicated for one minute, and then centrifuged at 15,000 × g for 10 min. A 50-μL clear supernatant of each sample was directly injected into the HPLC-ESI/MS/MS system. HPLC separations were performed using an Agilent HPLC 1200 pump (G1311A and micro vacuum degasser, G1322A), equipped with a thermostatted (set at 4 °C) well-plate autosampler (G1329A), linked to a 3200 QTAP mass spectrometer (from Applied Biosystems/MDS Sciex). Only the eluate fraction of 8-oxodG was delivered into the spectrometer, others were diverted to waste. All data were acquired and processed by Analyst^®^ Software 1.4.2 (MDS Inc., Concord, ON, Canada). Creatinine concentrations in diluted urine were measured at 505 nm by a Roche/Hitachi 902 Analyzer (Roche Diagnostics GmbH, Mannheim, Germany) using the Jaffe method with rate-blanked and compensated. Urine 8-oxo-7,8-dihydro-2′-deoxyguanosine results were expressed relative to creatinine concentrations. The coefficient of variation for this assay was 4.5%.

### Histological studies

Harvested kidneys were dissected into multiple sagittal and transverse sections for histological analysis. Sections were fixed in 10% formaldehyde in phosphate-buffered saline and embedded in paraffin. Paraffin sections were stained with haematoxylin-eosin (HE) for the presence of DHA crystals, calcification, inflammatory cells (including foreign body giant cells) and Masson’s Trichrome for fibrosis. The histological kidney sections were examined in a blinded fashion by the author (AKL) using light microscope (Olympus BX43, Notting Hill, Victoria, Australia) under total 100× magnification power with a polarizer for detection of DHA crystals. The severity of each morphological features were graded from 0 to +++ and the histological scores for adenine-treated animals were assessed in a semi-quantitated fashion (see statistical analysis).

### Statistical analysis

Statistical analysis was performed using SigmaPlot software (version 11.0). All values are expressed as mean ± standard deviation. All data were tested for normality and equality of variance, with appropriate parametric or non-parametric statistical tests applied. Two-tailed, unpaired t-tests (Student’s t test) or Rank Sum tests were applied for pairwise comparisons. Two way repeated-measures ANOVA was used to compare the differences between groups over time. Bonferroni post-hoc tests were applied to determine at which time-points significant differences between groups occurred. The Fisher exact test was used to determine differences in histological grading between Gunn and Wistar animals. A *P* < 0.05 was considered statistically significant.

## Additional Information

**How to cite this article**: Boon, A.-C. *et al.* Endogenously elevated bilirubin modulates kidney function and protects from circulating oxidative stress in a rat model of adenine-induced kidney failure. *Sci. Rep.* **5**, 15482; doi: 10.1038/srep15482 (2015).

## Supplementary Material

Supplementary Information
