# Supplementary material for: Endogenously elevated bilirubin modulates kidney function and protects from circulating oxidative stress in a rat model of adenine-induced kidney failure
Source: Sci Rep. 2015 Oct 26;5:15482. doi: 10.1038/srep15482 (PMC4620450; doi:10.1038/srep15482)
Supplement: Supplementary Information [file srep15482-s1.doc]

**Endogenously elevated bilirubin modulates kidney function and protects from circulating oxidative stress in a rat model adenine-induced kidney failure**

Ai-Ching Boon[1](http://www.sciencedirect.com/science/article/pii/S0891584912001736#aff1), Alfred K. Lam^2,3^, Vinod Gopalan^2^, Iris F. Benzie^4^, David Briskey^5^, Jeff S. Coombes^5^, Robert G. Fassett^5^ and [Andrew C. Bulmer](http://www.sciencedirect.com/science/article/pii/S0891584912001736)[1](http://www.sciencedirect.com/science/article/pii/S0891584912001736#aff1)*

^1^Heart Foundation Research Centre, Menzies Health Institute Queensland, Griffith University, Gold Coast, Australia.

^2^School of Medicine and Cancer Molecular Pathology, Menzies Health Institute Queensland, Griffith University, Gold Coast, Australia.

^3^Pathology Queensland, Gold Coast University Hospital, Gold Coast, Australia.

^4^Department of Health Technology & Informatics, The Hong Kong Polytechnic University, Hong Kong.

^5^School of Human Movement and Nutrition Sciences, University of Queensland, St Lucia, Australia.

*Andrew C. Bulmer

Heart Foundation Research Centre

Menzies Health Institute Queensland

Griffith University

Gold Coast, 4222

Australia

Email: a.bulmer@griffith.edu.au

*Corresponding Author

**Supplementary materials**

**Figure S1**. Effects of methylcellulose on polyuria and polydipsia. Urine output (a) and water consumption (b) in animals treated with methylcellulose for 10 days and monitored for 28 days (○, GC, n=9; □, WC, n=6). Data are expressed as a mean±standard deviation.

|  | **GC** | **WC** |
| --- | --- | --- |
| **HE** | **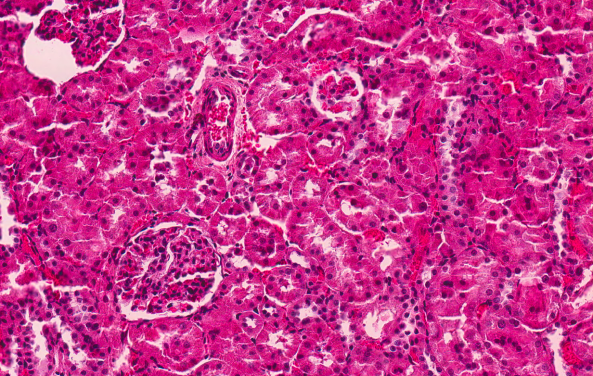** | 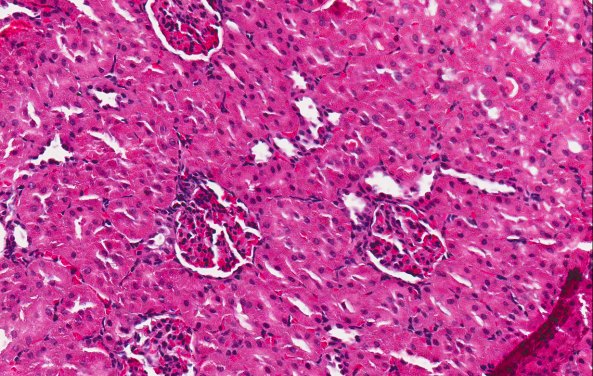 |

**Figure S2.** Effects of methylcellulose administration on kidney morphology. Histopathological examination of kidney sections stained with hematoxylin-eosin (HE) and in animals treated methylcellulose (GC and WC) at day 28. Kidney morphology appeared normal and therefore Masson Trichrome’s staining was not performed on these animals.

| **Blood biochemistry** | | | | | | | | | | | | | |
| --- | --- | --- | --- | --- | --- | --- | --- | --- | --- | --- | --- | --- | --- |
| **Day** | **-10** | | **0** | | | | | **14** | | | **28** | | |
| **Group** | **GA** | **WA** |  | **GA** | **WA** |  | **GA** | | **WA** |  | | **GA** | **WA** |
| **Albumin (g/L)** | 42.3±0.96 | 42.3±2.15 |  | 36.1±3.29 | 35.9±3.05 |  | 38.7±2.13 | | 37.8±2.58 |  | | 36.4±1.88 | 36.5±2.18 |
| **Total protein (g/L)** | 53.3±1.31 | 53.9±2.24 |  | 51.3±2.61 | 52.9±3.06 |  | 54.0±3.61 | | 53.9±1.68 |  | | 48.8±3.46 | 49.5±2.86 |
| **Glucose (mM)** | 12.7±1.65 | 12.7±2.3 |  | 11.8±2.75 | 11.3±0.94 |  | 11.6±1.52 | | 12.3±1.65 |  | | 11.2±3.7 | 10.9±4.12 |
| **Lipid profile** | | | | | | | | | | | | | |
| **Total cholesterol (mM)** | 1.26±0.11 | 1.48±0.14** |  | 1.67±0.19 | 1.76±0.26 |  | 1.64±0.18 | | 1.84±0.23** |  | | 1.29±0.16 | 1.45±0.15* |
| **Triglycerides (mM)** | 2.02±0.57 | 1.57±0.36** |  | 1.28±0.3 | 1.12±0.39 |  | 1.58±0.43 | | 1.52±0.58 |  | | 1.06±0.38 | 0.83±0.35 |
| **Lipase (U/L)** | 9.96±0.74 | 9.85±0.44 |  | 10.3±0.36 | 10.3±0.41 |  | 12.2±2.74 | | 11.5±2.34 |  | | 10.1±0.79 | 10.5±0.63 |
| **Liver function** | | | | | | | | | | | | | |
| **ALT (U/L)** | 51.8±6.6 | 48.8±7.46 |  | 179±120 | 72.1±55.8** |  | 62.9±24.0 | | 56±25.6 |  | | 45.9±10.3 | 38.3±5.92 |
| **AST (U/L)** | 79.8±24.0 | 104±60.1 |  | 202±90 | 158±81.0 |  | 185±251 | | 148±223 |  | | 68±14.5 | 66.4±14.1 |
| **GGT (U/L)** | 0.01±0.03 | 0.06±0.17 |  | 1.52±1.52 | 3.95±1.67** |  | 0.30±0.37 | | 0.25±0.39 |  | | 0.08±0.23 | 0.10±0.16 |

**Table S1.** Serum and urine biochemistries of adenine-treated Gunn and Wistar rats (n=12 per group).

Data are expressed as mean±standard deviation. **P*<0.05, ***P*<0.01 between the GA and WA groups.

**Table S2. Serum and urine biochemistries of methylcellulose-treated Gunn (n=9) and Wistar rats (n=6).**

| **Blood biochemistry** | | | | | | | | | | | | | | | | | |
| --- | --- | --- | --- | --- | --- | --- | --- | --- | --- | --- | --- | --- | --- | --- | --- | --- | --- |
| **Day** | **-10** | | **0** | | | | | | | | **14** | | | **28** | | | |
| **Group** | **GC** | **WC** |  | **GC** | **WC** | | |  | | **GC** | | **WC** |  | | **GC** | **WC** | |
| **Albumin (g/L)** | 41.7±3.23 | 42.1±2.98 |  | 43.5±2.74 | 43.5±3.56 | | |  | | 40.5±3.23 | | 41.2±1.54 |  | | 40.4±3.45 | 38.4±3.65 | |
| **Total protein (g/L)** | 53.1±3.82 | 54.9±3.87 |  | 55.3±3.3 | 54.4±1.75 | | |  | | 56.2±2.52 | | 54.6±2.17 |  | | 51.3±1.84 | 49.8±1.23 | |
| **Glucose (mM)** | 13.3±2.18 | 12.9±1.65 |  | 13.2±1.17 | 13.2±1.00 | | |  | | 12.1±2.58 | | 14.3±1.29 |  | | 10.5±3.47 | 9.7±0.73 | |
| **Lipid profile** | | | | | | | | | | | | | | | | | |
| **Total cholesterol (mM)** | 1.24±0.06 | 1.58±0.24** |  | 1.16±0.12 | 1.38±0.13* | | |  | | 1.18±0.1 | | 1.26±0.1 |  | | 1.15±0.34 | 1.15±0.06 | |
| **Triglycerides (mM)** | 1.53±0.67 | 1.55±0.09 |  | 1.72±0.43 | 1.23±0.25** | | |  | | 1.1±0.48 | | 1.2±0.51 |  | | 0.99±0.36 | 0.88±0.28 | |
| **Lipase (U/L)** | 10.4±1.58 | 9.72±0.31 |  | 9.93±0.42 | 9.96±0.41 | | |  | | 13.5±4.39 | | 9.62±0.54* |  | | 7.3±4.15 | 8.05±3.96 | |
| **Liver function** | | | | | | | | | | | | | | | | | |
| **ALT (U/L)** | 49.8±9.94 | 38.6±7.45 |  | 40.7±9.36 | 44.3±7.47 | | |  | | 40.9±3.42 | | 45.4±6.76 |  | | 42.3±11.2 | 47±18.2 | |
| **AST (U/L)** | 75.7±8.71 | 76.9±11.7 |  | 68.8±12.5 | 91.4±24.9** | | |  | | 491±8.59 | | 66.3±7.18 |  | | 61.1±6.05 | 64.5±16.4 | |
| **GGT (U/L)** | 0.06±0.17 | ND |  | 0.13±0.26 | ND | | |  | | 0.02±0.04 | | 0.08±0.2 |  | | ND | 0.03±0.05 | |
| **Kidney function** | | | | | | | | | | | | | | | | | |
| **Urea (mM)** | 5.89±1.06 | 7.27±1.69 |  | 6.12±0.96^#^ | 6.24±0.74^#^ | | |  | | 6.13±0.85^#^ | | 6.14±0.82^#^ |  | | 6.48±1.35^#^ | 6.25±0.72^#^ | |
| **Creatinine (µM)** | ND | ND |  | ND | ND | | |  | | ND | | ND |  | | ND | ND | |
| **Uric acid (µM)** | 54.2±9.16 | 57.3±18.4 |  | 57.3±24.2 | 53.5±20.7^#^ | | |  | | 47.7±11.1 | | 43.3±8.55 |  | | 69.2±45.2 | 48±7.77 | |
| **Calcium (mM)** | 2.4±0.29 | 2.34±0.22 |  | 2.54±0.14 | 2.66±0.14 | | |  | | 2.05±0.5 | | 2.48±0.23 |  | | 2.56±0.19 | 2.56±0.25 | |
| **Phosphate (mM)** | 2.55±0.14 | 2.64±0.12 |  | 2.18±0.39^#^ | 2.51±0.31^#^ | | |  | | 1.77±0.25 | | 2.10±0.11 |  | | 2.73±0.38 | 2.95±0.27 | |
| **24 hr urine excretion** | | | | | | | | | | | | | | | | | |
| **Urea (mmols)** | 130±66.2 | 172±67.2 |  | 127±61.2^#^ | | 123±67.9 |  | | 118±38.9^#^ | | | 143±27.2^#^ |  | | 146±65.5^#^ | | 183.7±73.5^#^ |
| **Creatinine (mmols)** | 7.86±4.08 | 9.96±2.84 |  | 9.15±4.90^#^ | | 8.75±4.99^#^ |  | | 7.71±3.10^#^ | | | 9.77±2.83^#^ |  | | 9.98±5.01^#^ | | 12.1±3.95^#^ |
| **Calcium (mmols)** | 0.86±0.72 | 0.53±0.45 |  | 0.39±0.25^#^ | | 0.46±0.25^#^ |  | | 0.50±0.38^#^ | | | 0.68±0.39^#^ |  | | 0.77±0.59^#^ | | 0.59±0.27^#^ |
| **Phosphate (mmols)** | 0.28±0.33 | 0.28±0.38 |  | 0.21±0.29^#^ | | 0.13±0.15 |  | | 0.09±0.12^#^ | | | 0.11±0.16^#^ |  | | 0.49±1.13 | | 0.11±0.13^#^ |
| **Total protein (mg)** | 0.18±0.11 | 0.22±0.09 |  | 0.55±0.12^#^ | | 0.33±0.10 |  | | 0.55±0.12^#^ | | | 0.33±0.10^#^ |  | | 0.55±0.12^#^ | | 0.33±0.10^#^ |

Data are expressed as mean±standard deviation. **P*<0.05, ***P*<0.01 between the GA and WA groups. ^#^*P*<0.05 between the adenine-treated and control groups.

| **24 hr electrolyte excretion** | | | | | | | | | | | |
| --- | --- | --- | --- | --- | --- | --- | --- | --- | --- | --- | --- |
| **Chloride (mmols)** | 22.6±12.4 | 22.5±9.03 |  | 29.5±16.3^#^ | 28.5±14.2 |  | 24.0±10.5^#^ | 27.8±12.8^#^ |  | 32.1±14.8^#^ | 35.2±17.5^#^ |
| **Potassium (mmols)** | 27.5±14.5 | 34.7±14.6 |  | 18.6±10.4^#^ | 19.8±11.0 |  | 17.4±5.92^#^ | 21.4±10.0^#^ |  | 22.0±10.2^#^ | 30.8±12.5^#^ |
| **Sodium (mmols)** | 8.91±5.74 | 6.68±6.49 |  | 6.99±3.84^#^ | 7.24±3.76 |  | 5.83±2.37^#^ | 6.52±3.22^#^ |  | 7.61±4.04^#^ | 9.73±5.37^#^ |

**Table S3.** Antioxidant and oxidative stress status in adenine-treated Gunn and Wistar rats (n=12 per group).

| **Antioxidants** | | | | | | | | |
| --- | --- | --- | --- | --- | --- | --- | --- | --- |
| **Day** | **-10** | | **0** | | **14** | | **28** | |
| **Group** | **GA** | **WA** | **GA** | **WA** | **GA** | **WA** | **GA** | **WA** |
| **Direct bilirubin (µM)** | 8.78±2.01 | 0.94±0.24** | 24.5±24.8 | 0.47±0.17** | 9.59±4.25 | 0.8±0.14** | 6.01±1.28 | 0.85±0.13** |
| **Total bilirubin (µM)** | 54.3±11.6 | 1.35±0.45** | 54.8±19.9 | 1.39±0.56** | 60.2±12.5 | 1.18±0.5** | 70.4±8.85 | 1.26±0.52** |
| **Reduced thiols (µM)** | 357±27 | 378±33 | 311±68 | 347±66 | 381±36 | 348±40 | 401±22 | 409±25 |
| **Oxidative stress/damage biomarkers** | | | | | | | | |
| **GSSG(µM)** | 6.11±1.79 | 6.20±1.84 | 5.23±1.48 | 4.89±2.50 | 4.83±1.75 | 3.92±0.71 | 4.03±1.90 | 4.08±1.41 |
| **GSH:GSSG** | 0.88±0.44 | 0.88±0.50 | 0.88±0.34 | 0.88±0.41 | 0.92±0.18 | 0.98±0.19 | 0.85±0.23 | 0.75±0.18 |
| **Urine**  **8-oxodG (nmol/nmol creatinine)** | 1.55±0.22 | 1.61±0.31 | 1.83±0.82 | 1.40±0.32 | 3.25±1.09 | 2.99±1.02 | 2.19±0.52 | 2.18±0.51 |

Data are expressed as mean±standard deviation. **P*<0.05, ***P*<0.01 between the GA and WA groups.

**Table S4.** Antioxidant and oxidative stress status in methylcellulose-treated Gunn (n=9) and Wistar rats (n=6).

| **Antioxidants** | | | | | | | | |
| --- | --- | --- | --- | --- | --- | --- | --- | --- |
| **Day** | **-10** | | **0** | | **14** | | **28** | |
| **Group** | **GC** | **WC** | **GC** | **WC** | **GC** | **WC** | **GC** | **WC** |
| **Direct bilirubin (µM)** | 9.71±2.57 | 0.72±0.18** | 6.56±1.97 | 0.9±0.19** | 6.51±2.16 | 1.37±1.05** | 6.13±0.77 | 0.88±0.27** |
| **Total bilirubin (µM)** | 53.4±16.8 | 0.9±0.34** | 92.8±28.8 | 1.42±0.47** | 85.3±8.56 | 1.73±1.2** | 92.1±9.5 | 1.57±0.16** |
| **GSH (µM)** | 4.96±2.69 | 5.44±3.30 | 3.51±0.68 | 3.06±0.66 | 4.36±2.40 | 3.18±0.77 | 2.69±0.88 | 3.00±0.56 |
| **Reduced thiols (µM)** | 353±56 | 363±26 | 471±72 | 443±53 | 393±48 | 413±17 | 392±42 | 435±47 |
| **Reduced thiols (nmols/mg protein)** | 6.69±1.12 | 6.65±0.83 | 8.49±1.08 | 8.15±0.85 | 6.93±0.90 | 7.56±0.38 | 7.63±0.67 | 8.74±0.88* |
| **Oxidative stress** | | | | | | | | |
| **GSSG (µM)** | 6.35±1.52 | 6.27±1.90 | 4.27±1.63 | 5.66±2.45 | 3.25±0.75 | 3.13±0.23 | 3.23±0.92 | 3.55±0.64 |
| **GSH:GSSG** | 0.86±0.48 | 0.96±0.57 | 0.90±0.31 | 0.63±0.30 | 1.35±0.74 | 1.02±0.26 | 0.84±0.17 | 0.86±0.18 |
| **Protein carbonyl (nmols/mg protein)** | - | - | - | - | 0.24±0.03 | 0.24±0.02 | 0.26±0.03 | 0.26±0.02 |

Data are expressed as mean±standard deviation. **P*<0.05, ***P*<0.01 between the GA and WA groups.

**Table S5. Histological evaluation of DHA crystal formation, inflammation, calcification, giant cells, inflammatory cells and fibrosis in adenine-treated animals (n=12 per group).**

|  | **HE staining** | | | | | **MT staining** |
| --- | --- | --- | --- | --- | --- | --- |
| **Group** | **DHA** | **Inflammation** | **Calcification** | **Giant cells** | **Inflammatory cells** | **Fibrosis** |
| **Gunn-adenine** | | | | | | |
| **Case** |  |  |  |  |  |  |
| 1 | +++ | + | ++ | +++ | + | + |
| 2 | +++ | + | ++ | +++ | + | + |
| 3 | + | + | ++ | + | + | + |
| 4 | +++ | + | 0 | +++ | + | + |
| 5 | +++ | + | + | +++ | + | + |
| 6 | +++ | + | 0 | +++ | + | + |
| 7 | +++ | + | ++ | +++ | + | + |
| 8 | +++ | + | + | +++ | + | + |
| 9 | +++ | + | + | +++ | + | + |
| 10 | +++ | + | ++ | +++ | + | + |
| 11 | +++ | + | 0 | +++ | + | + |
| 12 | +++ | + | + | +++ | + | + |
| **Wistar-adenine** | | | | | | |
| 1 | +++ | + | ++ | +++ | + | + |
| 2 | +++ | + | 0 | +++ | + | + |
| 3 | +++ | + | 0 | +++ | + | + |
| 4 | +++ | ++ | 0 | +++ | + | + |
| 5 | +++ | + | + | +++ | + | + |
| 6 | +++ | + | 0 | +++ | + | + |
| 7 | +++ | + | 0 | +++ | + | ++ |
| 8 | +++ | ++ | 0 | +++ | + | + |
| 9 | +++ | + | + | +++ | + | ++ |
| 10 | +++ | + | 0 | +++ | + | + |
| 11 | 0 | ++ | 0 | +++ | + | + |
| 12 | 0 | + | ++ | +++ | + | + |
| *P-*value | 1 | 0.217 | 0.371 | 1 | 1 | 0.478 |

**Table S6.** Histological evaluation of DHA crystal formation, inflammation, calcification, giant cells, inflammatory cells and fibrosis in methycellulose-treated animals (GC, n=9; WC, n=6).

| **HE staining** | | | | | |
| --- | --- | --- | --- | --- | --- |
| **Group** | **DHA** | **Inflammation** | **Calcification** | **Giant cells** | **Inflammatory cells** |
| **Gunn-methylcellulose** | | | | | |
| **Case** |  |  |  |  |  |
| 1 | 0 | 0 | 0 | 0 | 0 |
| 2 | 0 | 0 | 0 | 0 | 0 |
| 3 | 0 | 0 | 0 | 0 | 0 |
| 4 | 0 | 0 | 0 | 0 | 0 |
| 5 | 0 | 0 | 0 | 0 | 0 |
| 6 | 0 | 0 | 0 | 0 | 0 |
| 7 | 0 | 0 | 0 | 0 | 0 |
| 8 | 0 | 0 | 0 | 0 | 0 |
| 9 | 0 | 0 | 0 | 0 | 0 |
| **Wistar-methylcellulose** | | | | | |
| 1 | 0 | 0 | 0 | 0 | 0 |
| 2 | 0 | 0 | 0 | 0 | 0 |
| 3 | 0 | 0 | 0 | 0 | 0 |
| 4 | 0 | 0 | 0 | 0 | 0 |
| 5 | 0 | 0 | 0 | 0 | 0 |
| 6 | 0 | 0 | 0 | 0 | 0 |
